# Protocol for the Isolation and Analysis of Extracellular Vesicles From Peripheral Blood: Red Cell, Endothelial, and Platelet-Derived Extracellular Vesicles

**DOI:** 10.21769/BioProtoc.5487

**Published:** 2025-11-05

**Authors:** Bhawani Yasassri Alvitigala, Eranga Sanjeewa Wijewickrama, Laura Denney, Praveen Weeratunga, Pradeep Kaluarachchi, Ariaranee Gnanathasan, Lallindra Viranjan Gooneratne

**Affiliations:** 1 Department of Pathology, Faculty of Medicine, University of Colombo, Colombo, Sri Lanka; 2 Department of Clinical Medicine, Faculty of Medicine, University of Colombo, Colombo, Sri Lanka; 3 School of Clinical and Experimental Sciences, Faculty of Medicine, University of Southampton, Southampton, UK; 4 Healthcare Division, A. Baurs & Co. (Pvt.) Ltd., Colombo, Sri Lanka

**Keywords:** Bead-Free, Endothelial, Extracellular vesicles, Flow cytometry, Platelet, Red cell, Whole blood

## Abstract

This protocol describes the isolation and flow cytometric analysis of extracellular vesicles (EVs) derived from red blood cells, endothelial cells, and platelets in human peripheral blood. The protocol includes steps for preparing platelet-free plasma, fluorescent antibody staining, gating strategies, and technical controls. This protocol was developed within a study on EV release in snakebite-associated thrombotic microangiopathy; the protocol addresses challenges such as variable autofluorescence and heterogeneity in EV origin. It is flexible and can be adapted for alternative antibody panels targeting different cell populations or EV subtypes, including leukocyte-derived EVs.

Key features

• Bead-free, two-step plasma preparation enhances extracellular vesicle yield, reduces platelet contamination, and improves purity compared with conventional isolation methods for small-volume clinical samples.

• Reduced autofluorescence by compensation strategy using flow cytometry.

• Gating strategies to detect distinct EV populations derived from red cells, endothelial cells, and platelets.

• Validated in healthy donors and patients, enabling reproducible detection of EVs with broad downstream compatibility for flow cytometric applications.

## Graphical overview


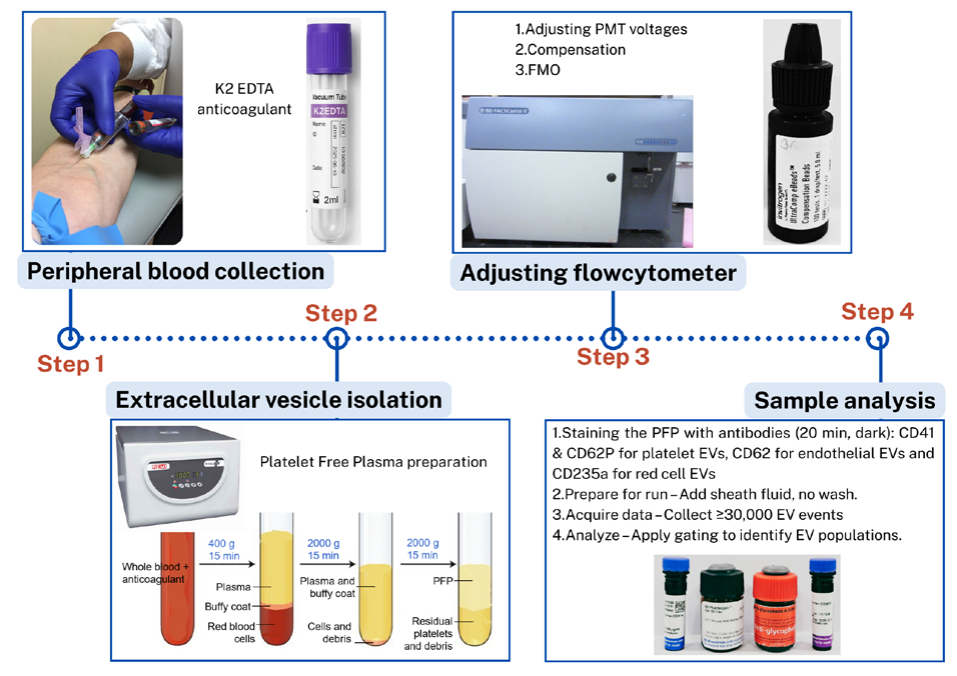


**Steps in the isolation and flow cytometric analysis of extracellular vesicles (EVs) derived from red blood cells, endothelial cells, and platelets in human peripheral blood**

## Background

This protocol outlines detailed steps for isolating extracellular vesicles (EVs) from human peripheral blood and characterizing them using flow cytometry [1]. It was developed as part of a study investigating EV release in patients with snakebite-associated thrombotic microangiopathy.

EVs play important roles in health and disease, but their small size, heterogeneity, and variable autofluorescence make analysis challenging [2]. Several techniques have been developed for EV analysis, including electron microscopy, nanoparticle tracking analysis, tunable resistive pulse sensing, western blotting, and ELISA-based assays, each with advantages and limitations [1]. Existing EV isolation protocols, including ultracentrifugation, precipitation, and size-exclusion chromatography, often face challenges such as incomplete removal of contaminating platelets, co-isolation of protein aggregates, variable EV recovery, and loss of vesicles during multiple washing steps [3]. Moreover, differences in centrifugation speeds, buffer preparation, and gating strategies limit reproducibility across laboratories [4]. Flow cytometry–based detection adds further complexity due to EV heterogeneity, variable autofluorescence, and the difficulty of distinguishing true EVs from debris [5].

Bead-based analysis of EVs is commonly used to overcome the challenge of their small size, which limits direct detection by conventional flow cytometry. In this approach, EVs are captured onto larger beads (typically 4–6 μm) coated with antibodies against tetraspanins such as CD63, CD81, or CD9, thereby increasing their effective size and enabling detection and characterization by flow cytometry. Once bound, the bead–EV complexes can be further probed with fluorescent antibodies to analyze additional surface markers and identify subpopulations. While this method is practical and widely accessible, it has important limitations: it selectively enriches only those EVs expressing the capture antigen (e.g., CD63^+^ vesicles if anti-CD63 beads are used), potentially overlooking other populations, and multiple EVs may attach to a single bead, preventing true single-vesicle resolution. Consequently, bead-based analysis provides useful phenotyping information but does not fully represent the heterogeneity of circulating EVs [6,8].

Our protocol addresses these issues by providing a standardized platelet-free plasma preparation method to minimize platelet contamination, with no unnecessary washing steps to reduce EV loss, use of filtered buffers to decrease background noise, and detailed stepwise explanations with troubleshooting guidance to improve reproducibility. Importantly, the protocol is a bead-free, flexible approach that can be adapted for different antibody panels and EV subtypes, enhancing its applicability compared with many existing protocols.

## Materials and reagents

**Biological materials**

1. Human whole blood samples

*Note: This study was approved by the Ethics Review Committee of the Faculty of Medicine, University of Colombo, Sri Lanka, EC-22-080).*

**Reagents**

1. Mouse anti-human glycophorin A [GAR-2(HIR-2)] or CD235a, PE (25 μg/mL) (BD Biosciences, catalog number: 340947)

2. Mouse anti-human CD62E (68-5H11), APC (25 μg/mL) (BD Biosciences, catalog number: 551144)

3. Mouse anti-human CD62P (AK-4), BV421 (25 μg/mL) (BD Biosciences, catalog number: 564038)

4. Mouse anti-human CD41a (HIP8), PE-Cy^TM^7 (200 μg/mL) (BD Biosciences, catalog number: 5641424)

5. UltraComp eBeads^TM^ compensation beads (ThermoFisher Scientific, catalog number: 01-2222-41)

6. BD FACSDiva CS&T research beads (BD Biosciences, catalog number: 655050)

7. BD OneFlow setup beads (BD Biosciences, catalog number: 658620)

8. FACS clean solution, 5 L (BD Biosciences, catalog number: 340345)

9. FACS shutdown solution, 5 L (BD Biosciences, catalog number: 334224)

10. FACS flow sheath, 20 L (BD Biosciences, catalog number: 342003)

11. Sodium chloride (NaCl)

12. Potassium chloride (KCl)

13. Disodium hydrogen phosphate, anhydrous (Na_2_HPO_4_)

14. Potassium dihydrogen phosphate (KH_2_PO_4_)

15. Phosphate buffer saline (1×), pH 7.4 (Thermo Fisher Scientific, catalog number: 10010023)

**Laboratory supplies**

1. Plastic round bottom tubes 3 mL (Plastica International Pvt. Ltd., Sri Lanka)

2. Falcon^®^ round bottom polystyrene tubes (FACS tubes) (Corning Life Sciences, catalog number: 352054)

3. Micropipette tips: 5 μL, 200 μL, 1,000 μL (BIOLOGIX, China)

4. Microcentrifuge tube (1.5 mL) (BIOLOGIX, China)

5. 3 mL disposable syringe with 23-gauge needle (Changzhou Medical appliances, China)

6. K2-EDTA vacutainer tubes [CML biotech (Pvt) Ltd., Sri Lanka]

7. Filters with pore size 0.2 μm

## Equipment

1. BD FACSCanto^TM^ II Flow Cytometer (BD Biosciences, USA, catalog number: R33896203257)

2. REMI R-8C PLUS centrifuge (Remi Elektrotechnik Ltd., India, catalog number: ZGEN-10144)

3. Vortex mixer (Remi Elektrotechnik Ltd., India)

4. Refrigerator (2–8 °C)

## Software and datasets

1. BD FACSDiva Software (BD Biosciences, USA, version 8.0.3)

2. FlowJo^TM^ (BD Biosciences, USA, version 10.0)

## Procedure

**A. Instrument setup and calibration (Timing: 1 h)**

1. Set up the flow cytometer (see Table 1) and perform thorough cleaning of the slit, flow cell, and flow tubes by following the cleaning mode (using FACS clean solution and FACS flow sheath).

2. Ensure proper calibration using BD OneFlow setup beads and FACSDiva CS&T research beads.

a. Mix one drop of BD OneFlow setup beads in 500 μL of sheath fluid.

b. Mix one drop of FACSDiva CS&T research beads in another 500 μL of sheath fluid.

**Table 1.** Specifications of the flow cytometer used in the study

Target EV	^β^CD marker	Clone	Fluorochrome	Laser	Excitation wavelength	Filter set
Platelets	CD62P	AK-4	BV421	Violet (V1)	405 nm	450/50
Endothelial	CD62E	68-5H11	APC	Red (R1)	633 nm	660/20
Red blood cells	CD235a	HIR2	PE	Blue (B2)	488 nm	585/42
Platelet	CD41a	HIP8	PE-CY7	Blue (B4)	488 nm	780/60

**Instrument and software:** BD FACSCanto II with BD FACSDiva software version 10.2.3.

^β^The selected CD markers were specific for the type of cell intended for analysis [7,9–11].

EV: extracellular vesicles

Figure S1 provides the spectral view map [12].

**Critical:**

1. Store all reagents at the manufacturer’s recommended temperatures. Remove them from storage only when needed and return them immediately after use. Keep reagents away from direct light when handling and transferring, since fluorophores are photosensitive.

2. When analyzing multiple samples in a single run, it is important to run sheath fluid through the sample injection port (SIP) for at least 1 min between samples. While this is not necessary for cell-based analysis, we find that directly swapping between samples without this additional wash step resulted in cross-sample contamination, which confounded analysis of the subsequent sample, leading to false EV calculations or inaccurate results. This practice ensures that the analysis of each sample is independent and that any potential artifacts from the previous sample do not affect the analysis of the subsequent sample.

**B. Peripheral blood collection (timing: 10 min)**

*Note: This step describes the procedure for collecting peripheral whole blood samples from a patient for EV analysis.*

1. Collect 3 mL of peripheral blood using a 23-gauge needle and syringe.

2. Transfer blood to a K2-EDTA tube (commercially prepared dipotassium ethylenediaminetetraacetic acid anticoagulant tube) and invert gently 3–4 times until blood and K2-EDTA anticoagulant are well mixed.

3. Perform centrifugation for plasma separation within 4 h of blood collection.

*Notes:*

*1. Use of a large gauge needle prevents lysis of red blood cells. EDTA-anticoagulated whole blood is generally preferred for EV analysis in flow cytometry because it effectively prevents coagulation artifacts, stabilizes cells without releasing additional EVs, and minimizes platelet aggregation.*

*2. By chelating calcium, EDTA inhibits platelet activation and clot formation, which can otherwise lead to an artificial increase in EV numbers. Additionally, it helps maintain the integrity of EVs and reduces the risk of EV aggregation. Alternatively, whole blood can be collected in tubes containing 3.2% sodium citrate, particularly for coagulation studies related to platelet-derived EVs, since it preserves the physiological level of calcium [13]. The choice of anticoagulant should be guided by the specific objectives of the EV analysis and the need for standardization across studies.*

**Critical:**

1. Processing samples within 4 h for EV analysis in flow cytometry is crucial to ensure sample integrity and accurate results. EVs are highly dynamic and prone to degradation, fusion, or spontaneous release over time, especially due to ongoing cellular activation or apoptotic processes. Delayed processing can lead to artificial increases in EV counts, altered size distribution, and compromised marker expression, leading to unreliable data.

2. Typically, neutrophils can be prone to lysis when stored or transported on ice; however, when working in higher ambient temperatures (>25 °C), it is advised to keep them on ice to slow down cellular metabolism and prevent additional vesicle shedding. Cooling the sample reduces enzymatic activity and prevents unwanted platelet activation, which can otherwise lead to secondary EV formation. However, freezing is generally avoided as it can cause vesicle rupture or aggregation. Proper handling and timely processing are essential for obtaining reproducible and biologically relevant EV data.

**C. Preparation of platelet-free plasma (PFP) (timing: 45 min)**

*Note: This section describes the procedure for platelet-free plasma (PFP) preparation for isolation of EVs from the whole blood sample.*

1. Centrifuge EDTA blood (2 mL) at 400× *g* for 15 min at room temperature.

2. Transfer supernatant carefully to the plastic round-bottom tube, leaving ~500 μL above the buffy coat so that the red cell pellet and the buffy coat with platelets and white cells are discarded.

3. Centrifuge at 2,000× *g* for 15 min at room temperature.

4. Transfer supernatant, leaving ~100 μL behind.

5. Perform a final centrifugation at 2,000× *g* for 15 min.

6. Aliquot the supernatant, leaving ~100 μL behind. EVs are now concentrated in the supernatant, which will be used for analysis. This supernatant is devoid of platelets and hence known as PFP [14].

**Critical:** The first centrifugation step at a lower speed (typically, 400× *g*) is performed to remove intact cells and large debris while preventing excessive mechanical stress that could lead to cell lysis and artificial EV release. If a higher speed were used initially, fragile cells, especially platelets, could rupture, artificially increasing EV counts and altering their composition. This low-speed centrifugation effectively separates whole cells from the plasma, ensuring that only naturally circulating EVs remain for subsequent isolation and analysis (Troubleshooting 1).

**Figure 1.**
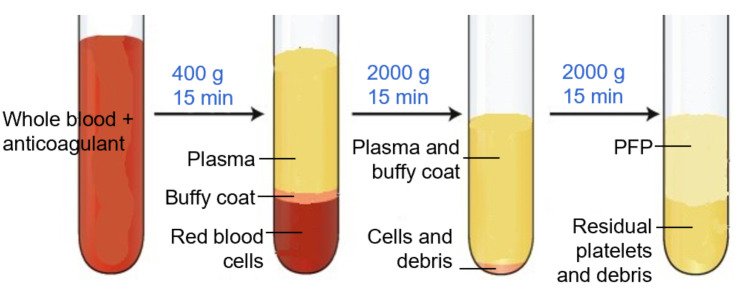
Platelet-free plasma (PFP) separation. PFP is the upper 100–200 μL layer, which is devoid of platelets, cell debris, and buffy coat.

*Note: At each step, new micropipette tips should be used to avoid contamination of cells in the PFP (Figure 1). Care should be taken when removing tubes from the centrifuge, as the layers are easily disturbed; unlike standard cell pellets, EV fractions do not form solid pellets and remain loosely associated with the supernatant (Troubleshooting 2).*

**D. Adjusting PMT voltages for sample analysis (timing: 1 h)**

*Note: This setup allows for enhanced resolution of small particles and minimizes the loss of relevant events due to inappropriate scatter thresholds.*

1. Use unstained PFP from a healthy donor (as a control, without any antibodies) to determine the baseline autofluorescence and scatter.

2. For the second tube, use unstained PFP from a healthy donor.

a. Transfer 100 μL of PFP into a 5 mL FACS acquisition tube.

b. Add 2 μL of each antibody (CD41A, CD62P, CD62E, CD235a) to the tube.

c. Incubate in the dark for 20 min at room temperature.

d. Add 500 μL of sheath fluid and mix gently.

**Figure 2.**
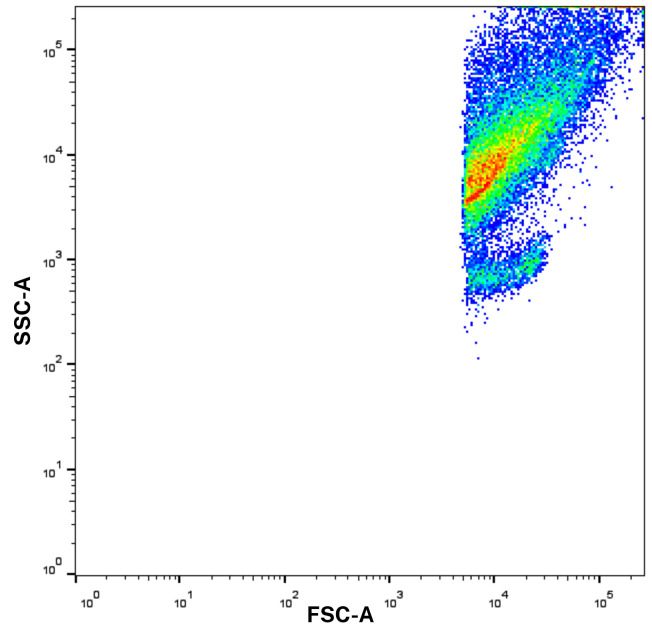
Forward scatter and side scatter (FSC/SSC) scale with logarithmic scaling before gating. Log scaling enhances the resolution of extracellular vesicles (EVs), which would otherwise be difficult to distinguish using standard linear settings.

3. To ensure accurate detection of EVs, it is essential to optimize the forward scatter (FSC) and side scatter (SSC) settings of the flow cytometer. Standard flow cytometry settings are typically optimized for whole cells ranging from 5 to 10 μm in size. In contrast, EVs are considerably smaller (typically, 100–1,000 nm) and exhibit variable internal complexity, making them challenging to resolve with conventional FSC/SSC voltages.

4. Therefore, prior to sample acquisition, the flow cytometer should be set to high sensitivity mode. FSC and SSC voltages should be adjusted appropriately to reduce noise while still capturing small particles. Importantly, while cells are typically analyzed using linear FSC/SSC scales, switching to logarithmic scaling is essential for resolving the size differences present in EVs and other submicron particles. Calibration using size-standard beads (e.g., 100, 200, and 500 nm) is recommended to establish the detection range and gate the EV population accurately (Figure 2).

5. Voltages are adjusted first to optimize signal detection and ensure that fluorescence intensities fall within the most sensitive parts of the dynamic range of the detector. Increase the voltage if the signal is too dim. Decrease the voltage if the signal is saturating or spilling over into other channels. Aim for optimal separation between negative and positive populations while keeping fluorescence within the linear range. Once voltages are set, they should remain unchanged for the entire experiment to ensure consistency.

6. Adjusted voltages for EVs in our experiment: FSC = 375 V; SSC = 373 V; APC-H7 = 558 V; BV421 = 416 V; PE = 432 V; APC = 508 V. These voltage settings are lower than those typically used for peripheral blood mononuclear cell (PBMC) analysis, reflecting the much smaller size of EVs. FSC and SSC voltages should be changed depending on the cytometer and the level of granularity. In contrast, detecting EVs (which are much smaller) requires significantly lower FSC/SSC thresholds.

**E. Compensation setup (timing: 20 min)**

*Note: This step is needed in flow cytometry to correct for spectral overlap between fluorochromes, ensuring accurate measurement of each fluorescent signal.*

1. Vortex compensation beads for 30 s.

2. Prepare four Falcon tubes (C1–C4) and add two drops of compensation beads for each tube.

3. Add 100 μL of protein-free phosphate buffer saline (PBS) to each tube.

4. Add 1 μL of one type of antibody (manufacturer’s suggested concentration) to each tube as follows:

a. Tube C1: Only 1 μL of CD235a antibody

b. Tube C2: Only 1 μL of CD41a antibody

c. Tube C3: Only 1 μL of CD62P antibody

d. Tube C4: Only 1 μL of CD62E antibody

*Note: When staining compensation beads, a much lower amount of antibody—typically 5%–10% of the amount recommended for staining cell populations—is usually sufficient. However, it is important to optimize and titrate the antibodies carefully to ensure that the mean fluorescence intensity (MFI) of the positive peak on the compensation beads exceeds that of the stained samples.*

5. Mix well and incubate all the tubes in the dark at room temperature for 10 min.

6. Wash with PBS and centrifuge at 800× *g* for 5 min.

*Note: Beads can be centrifuged at much higher speeds than typical for cells or EVs.*

7. Resuspend in 200 μL of PBS and perform compensation in BD FACSCanto II.

*Note: In flow cytometry, the compensation step is necessary to correct for fluorescence spillover, which occurs when a fluorophore emits light that is detected in multiple channels due to spectral overlap. Compensation ensures that each detector measures only the intended fluorescence signal by mathematically subtracting the contribution of spillover from other fluorophores.*

**Critical:** Non-isotonic solutions, such as water, are unsuitable for sample dilution because their hypotonic nature disrupts the osmotic balance required to preserve the integrity of cells and EVs. If cells and EVs are exposed to non-isotonic solutions, i.e., water, osmosis pressure causes swelling and eventual lysis (bursting) due to the difference in osmotic pressure. Instead, isotonic solutions like PBS, sheath fluid, or any commercially available flow cytometry buffers can be used. These solutions maintain the osmotic balance, preventing cell and EV lysis while ensuring optimal flow and signal detection in the cytometer.

**F. Fluorescence-minus-one (FMO) control setup (timing: 25 min)**

*Note: FMO is essential for appropriate gating and data interpretation in flow cytometry. They are distinct from compensation controls in that they help in accurate gating by distinguishing true positive signals from background fluorescence and spillover effects in multi-color flow cytometry.*

1. Prepare five tubes containing PFP and respective antibodies (Table 2).

**Table 2.** Preparation of five tubes for FMO

Tubes		All	FMO 1	FMO 2	FMO 3	FMO 4
Plasma sample		100 μL	100 μL	100 μL	100 μL	100 μL
Antibody	CD41a	2 μL	-	2 μL	2 μL	2 μL
	CD235a	2 μL	2 μL	-	2 μL	2 μL
	CD62P	2 μL	2 μL	2 μL	-	2 μL
	CD62E	2 μL	2 μL	2 μL	2 μL	-
FMO, fluorescence-minus-one						

2. Incubate for 20 min at room temperature (dark).

3. Add 500 μL of sheath fluid.

4. Acquire at least 30,000 EV events using BD FACSCanto II. Maintain a sample flow rate at a medium setting with a FACS flow pressure of 2.83 × 10^4^ Pa.

5. Using BD FACSDiva Software (v8.0.3):

a. Apply SSC-A vs. FSC-A gating for EV population identification based on their size and granularity.

b. Perform FSC-A vs. FSC-H gating to exclude doublets and isolate distinct populations like EVS.

*Notes:*

*1. It is important to note that no washing steps are included in the FMO control setup, as EVs are extremely small and can be lost during centrifugation and washing due to their low mass and fragility. Additionally, since we used carefully prepared PFP and filtered buffers, washing steps were not required. This helped us to preserve EV yield.*

*2. Buffers are filtered with 0.2 μm filters, which will reduce the debris in FSC and SSC that can be mistaken for EVs.*

*3. Unlike in conventional cell analysis, washing steps are often omitted in EV analysis. This is primarily to avoid sample loss, as EVs are small and fragile, and centrifugation can significantly reduce their yield. While cellular debris is largely removed during PFP preparation, it is important to acknowledge that unbound antibodies can still contribute to background signal or bind nonspecifically over time—particularly after fixation. To mitigate this without washing, we use minimal antibody volumes, as recommended by manufacturer guidelines, to limit excess unbound antibody while preserving EV detection sensitivity. This approach allows for a balance between reducing nonspecific binding and maintaining EV yield. (see Troubleshooting 4).*

*4. FMO controls (FMO 1–4 tubes) contain all fluorochromes in the panel except one. This missing fluorochrome allows assessing the performance of the compensation and background fluorescence in that specific channel due to spillover from other fluorochromes and autofluorescence, by comparing with the tube that contains all fluorochromes.*

**Critical:**

1. Total events set at 30,000 can be adjusted depending on the specific needs of the analysis. The event count is usually chosen to ensure that there are enough EVs or target events relative to non-EV objects for statistical accuracy. If you have a lower EV concentration relative to non-EV objects, you may need to increase the event count to capture a sufficient number of EVs for reliable analysis. Conversely, for higher EV concentrations, you might reduce the event count to avoid excessive data collection and to save time.

2. Care should be taken not to exceed the instrument’s recommended maximum events per second to ensure accurate data collection. If the number of events exceeds this, dilute the sample and re-acquire. Adjusting both event count and run-time can be optimized based on the sample's characteristics and the specific goals of your EV analysis.

**G. Gating and sample analysis (timing: 25 min)**

*Note: Gating is crucial for accurately identifying and quantifying specific cell populations within the sample.*

1. Transfer 100 μL of PFP into a 5 mL FACS acquisition tube.

2. Add 2 μL of each antibody (CD41A, CD62P, CD62E, CD235a) to the tube. (As EV yield is high in the PFP sample, based on the manufacturer's recommendations, 2 μL of antibody is adequate for optimal staining.)

3. Incubate in the dark for 20 min at room temperature (see Troubleshooting 3).

4. Add 500 μL of sheath fluid and mix gently. (To preserve EV yield, no washing steps are performed. Instead, the sample is diluted in sheath fluid to ensure smooth and consistent aspiration during flow cytometry.)

5. Acquire 30,000 positive events using BD FACSCanto II. Maintain a sample flow rate at a medium setting with a FACS flow pressure of 2.83 × 10^4^ Pa.

6. Using BD FACSDiva Software (v8.0.3).

a. Apply SSC-A vs. FSC-A gating for EV population identification based on their size and granularity.

b. Perform FSC-A vs. FSC-H gating to exclude doublets and isolate distinct populations like EVs.

*Note: The concentration or volume of a CD marker (antibody) used in flow cytometry is typically recommended by manufacturers for cell analysis, often based on 100 μL of sample or 1 × 10^6^ cells. However, EV analysis may require different optimal conditions due to their smaller size and lower antigen density; therefore, antibody titration should be performed specifically for EV work to ensure adequate signal and minimal nonspecific binding.*

**Critical:** EVs are generally very small and lack significant internal complexity. Due to minimal granularity, SSC-A (area) may not add significant value. The focus on FSC-A and FSC-H (height) helps in accurately sizing these small vesicles without introducing unnecessary complexity from SSC data.

Further off-instrument analysis of the EV populations can be done by FlowJo (v10.0).

## Data analysis

This protocol enables the relative quantification and characterization of red cell-, endothelial-, and platelet-derived EVs from human peripheral blood. This protocol was developed and validated in both healthy controls and patients with disease, specifically to assess EV release in snakebite-associated thrombotic microangiopathy following envenoming. Flow cytometry has previously been used to assess EV release in thrombotic disorders, including snakebite-associated thrombotic microangiopathy, demonstrating its clinical relevance [15].

**Figure 3.**
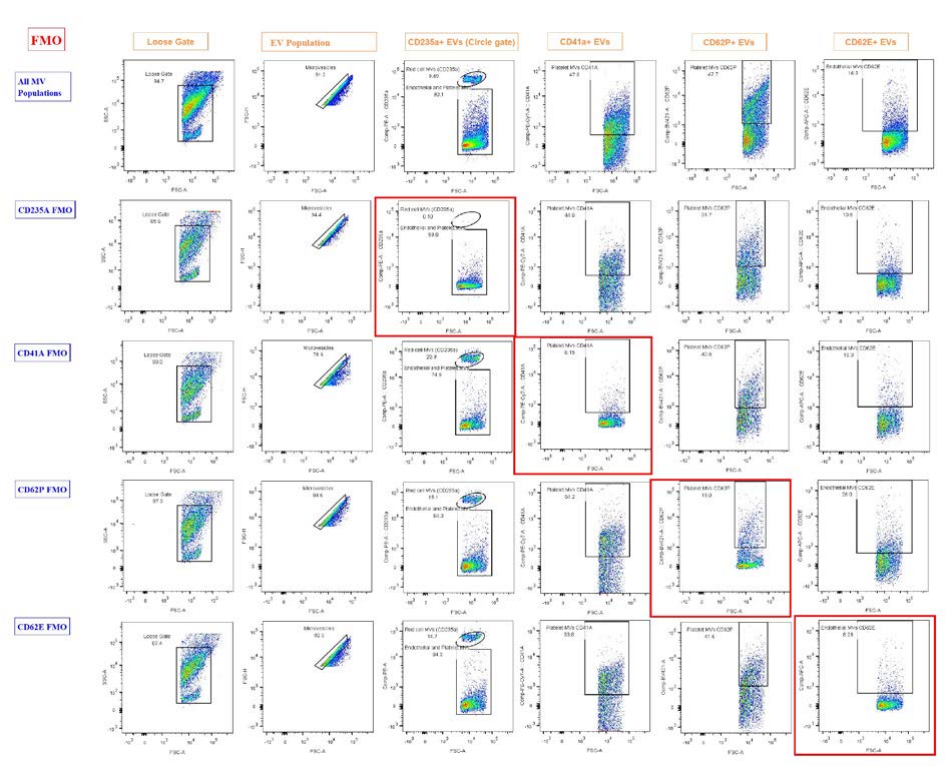
Results of fluorescence-minus-one (FMO). FMO controls were used to define gating boundaries for the identification of extracellular vesicle (EV) subpopulations based on cell-specific markers. Each row represents FMO controls for a specific marker, where one fluorochrome is omitted to determine background fluorescence and set gates accurately. The first row ("All EV Populations") displays the full staining panel, used to demonstrate the overall gating strategy and EV subpopulation distribution. Subsequent rows show FMOs for CD235a, CD41a, CD62P, and CD62E, with the marker of interest omitted in each row. Gating regions were drawn using the negative populations in FMO controls (highlighted by red boxes) to accurately identify positive events in fully stained samples. EVs have a highly diverse composition, with some containing abundant proteins or internal structures. This heterogeneity results in greater variability and often higher levels of intrinsic fluorescence, which was particularly evident in the CD62P FMO. Loose gating was observed in FCS-A vs. SSC-A and in microvesicles on FSC-A vs. FSC-H. Gating for CD235a, CD41a, CD62P, and CD62E was performed on the compensated axis vs. FSC-A.

Researchers can expect well-separated populations in fluorescence histograms, with accurate identification of EV subtypes using specific surface markers. The gating strategy ensures the exclusion of debris and doublets, providing high reproducibility (Figure 3). Flow cytometry plots should reveal distinct populations of CD41a^+^ platelet-derived EVs, CD62E^+^ endothelial EVs, and CD235a^+^ red cell–derived EVs. The use of FMO controls aids in defining positive and negative populations. This backbone of EV analysis can be expanded to include further markers of interest.

## Validation of protocol

This flow cytometry protocol for the analysis of EVs in blood has been used in an ongoing study of patients with snakebite-associated thrombotic microangiopathy. To ensure reproducibility and reliability of this protocol, we undertook several validation measures. FMO and controls were performed to accurately define EV populations. 20 healthy control samples were analyzed in batch to establish baseline MV isolation patterns in normal controls. In total, we examined 35 patient samples across different clinical presentations, and for each patient sample batch analyzed, a normal control was included in parallel.

All acquisitions were performed by the same researcher using the same flow cytometer (BD FACS Canto II) and standardized consumables (same batch of blood collection tubes, syringes, centrifuge, and reagents) to minimize both inter-operator and instrument-to-instrument variability. This standardized approach reduced potential technical variation and increased confidence in biological differences observed.

Statistical analyses were performed using GraphPad Prism/SPSS to compare EV counts among groups (healthy controls and patients), showing significant differences consistent with disease pathology. EV profiles of a control and a patient are included in Figure S2. Notably, endothelial EVs (CD62E^+^) and red cell EVs (CD235a^+^) were significantly elevated in snakebite patients compared to controls (median red cell EVs 63.2 vs. 26.2, p < 0.05; median endothelial EVs 40.6 vs. 4.3, p < 0.05), suggesting that endothelial injury plays a central role in its pathophysiology. These findings are currently being prepared for submission in a separate research manuscript.

Although the associated research article has not yet been published, the protocol has been applied in a clinical research study supported by the South Asian Clinical Toxicology Research Collaboration, Sri Lanka (SACTRC). The method has proven robust and reproducible in this context and will form the core of the methodology section in our upcoming publication.

## General notes and troubleshooting

**General notes**

1. In flow cytometry, selecting antibodies from various manufacturers is acceptable, provided they specifically target the intended antigens; however, variations in clone specificity and fluorochrome compatibility require careful validation to ensure consistent performance. Assigning unique fluorochromes to each antibody in a multicolor panel is crucial to prevent spectral overlap, and antibodies should be validated for flow cytometry applications to maintain assay reliability. Fluorophore antibody panels should be designed using your specific flow cytometer’s laser setup and filter combination, as well as online spectral analyzer tools used to assess spectral overlap, prevent fluorophore clash, and determine necessary compensation levels. Additionally, ensure that antibodies are within their effective usage period or best-before date and be vigilant about potential concentration variations between manufacturers [5,16].

2. Autofluorescence variability among samples can interfere with fluorescence-based gating, requiring careful optimization. Additionally, distinguishing EVs from cellular debris remains a challenge.

3. Carboxyfluorescein succinimidyl ester (CFSE) is a fluorescent, cell-permeable dye that covalently binds to amine groups of proteins. When used in EV studies, CFSE labels the vesicular protein content, allowing clear discrimination of true EVs from background noise, debris, or unbound antibodies during flow cytometric analysis. By providing a consistent fluorescent signal from the vesicle interior, CFSE can enhance the sensitivity and accuracy of EV detection, particularly for small EVs with low surface antigen density, improving overall reproducibility [17].

4. Minimal Information for Studies of Extracellular Vesicles (MISEV) compliance refers to adherence to the guidelines established by the International Society for Extracellular Vesicles (ISEV) to ensure rigor, reproducibility, and transparency in EV research. The 2018 MISEV guidelines recommend detailed reporting of EV sources, isolation methods, characterization using both positive (transmembrane/lipid-bound and cytosolic) and negative markers for contaminants, particle size and morphology, and experimental controls. Compliance ensures that EV studies provide sufficient information for other researchers to replicate findings and critically evaluate the validity of EV-related data [18,19].

5. The current protocol for isolating and analyzing EVs from peripheral blood is partially compliant with MISEV guidelines. It provides detailed steps for sample collection, platelet-free plasma preparation, flow cytometric staining, gating, and use of compensation and FMO controls. Positive markers for EV subtypes (platelet, endothelial, and red blood cell–derived) are included, and extensive troubleshooting guidance is provided.

6. This protocol provides a reproducible and robust approach for EV analysis; however, it has certain limitations. The detection of EVs is influenced by their small size and low refractive index, which may impact sensitivity and accuracy. The protocol does not incorporate complementary characterization techniques such as electron microscopy or nanoparticle tracking analysis, which are recommended for full MISEV compliance. Importantly, we aimed to develop a protocol that could be realistically implemented in resource-limited environments, thereby contributing to the democratization of scientific methods and access. Inclusion of CFSE staining or similar dyes, as suggested, could further enhance compliance by improving EV discrimination.

**Troubleshooting**

**Problem 1:** Loss of EVs or low event rate during acquisition.

Possible cause: Overly harsh centrifugation or additional washing steps may pellet or discard small EVs, reducing their yield in the final preparation.

Solution: Avoid centrifuging at speeds above 2,500× *g* and do not include unnecessary washing steps unless validated. Carefully aspirate the supernatant during the final steps, leaving ~100 μL behind to protect EV content from being lost with the pellet.

**Problem 2:** Visible debris or red tinge in the supernatant.

Possible cause: This typically occurs when the supernatant is collected too close to the buffy coat or red cell layer, leading to contamination with residual red blood cells or leukocytes.

Solution: During the initial centrifugation step, carefully aspirate the supernatant while leaving at least 500 μL above the buffy coat. Use a pipette rather than vacuum aspiration to maintain precision and avoid disturbing the cellular layers.

**Problem 3:** Clumps or visible particulates in the sample.

Possible cause: Slow or inconsistent sample handling, temperature changes, or inadequate mixing can lead to aggregation or precipitation.

Solution: Perform all centrifugation and handling steps at a consistent room temperature (25 °C). Process samples without prolonged pauses and gently mix plasma prior to each transfer to ensure homogeneity.

**Problem 4:** Increased background and non-zero % FMO controls.

Possible cause: Diverse composition of EVs.

Solution: Cells such as red blood cells and platelets contain fundamental components like nuclei, membrane proteins, lipids, and mitochondria, which contribute to a relatively consistent pattern of autofluorescence. In contrast, EVs have a highly diverse composition. Some may contain abundant protein or internal structures, while others contain very little. This heterogeneity results in more variable and often higher levels of innate fluorescence. A crucial step is the proper preparation of PFP. Maximum care should be taken to avoid transferring intact cells into the final aliquot. An increased background signal is not necessarily due to poor compensation. Instead, elevated autofluorescence is an expected feature of EV analysis, and a certain level of positive signal in FMO or no-antibody controls is typical and expected.

## Supplementary information

The following supporting information can be downloaded here:

1. Figure S1. Spectral view map

2. Figure S2. Snakebite-associated thrombotic microangiopathy patients demonstrated elevated red cell- and endothelial-derived EV populations compared with controls, reflecting venom-mediated endothelial injury
